# M2 middle cerebral artery dissection on 7T MRI

**DOI:** 10.1136/svn-2022-001557

**Published:** 2022-07-14

**Authors:** Xuewei Xie, Zhe Zhang, Qingle Kong, Hui Qu, Jing Jing

**Affiliations:** 1 Department of Neurology, Beijing Tiantan Hospital, Capital Medical University, Beijing, China; 2 China National Clinical Research Center for Neurological Diseases, Beijing, China; 3 Tiantan Neuroimaging Center of Excellence, Beijing, China; 4 MR Collaboration, Siemens Healthineers Ltd, Beijing, China

**Keywords:** Magnetic Resonance Imaging, dissection

**Figure 1:**
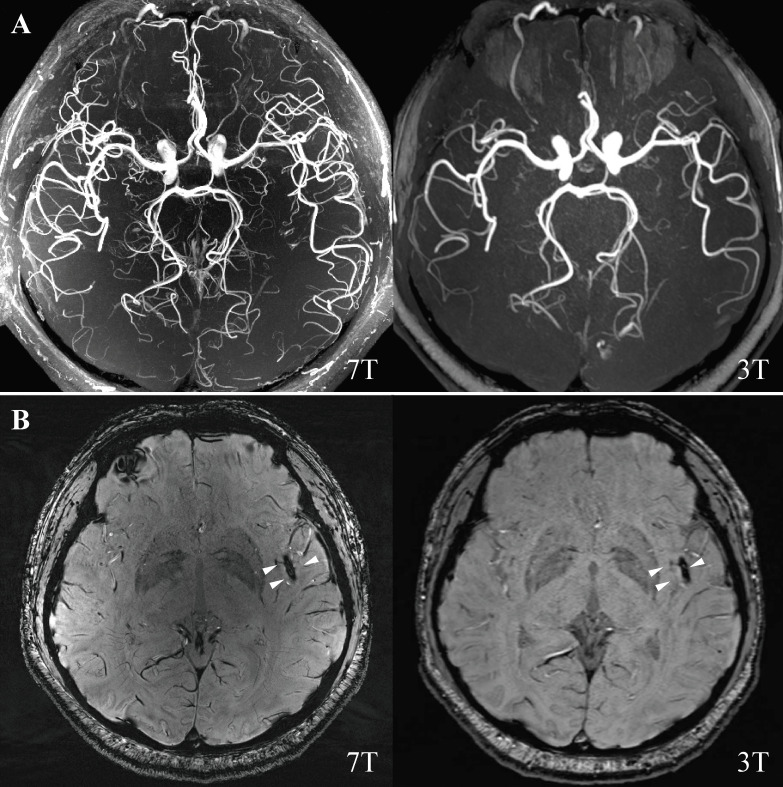
(A) 7T and 3T magnetic resonance angiography. (B) 7T and 3T SWI were showing blooming hypointense signals (susceptibility vessel sign) in the distal M2 segment of the left MCA (white triangle). MCA, middle cerebral artery; SWI, susceptibility-weighted imaging.

**Figure 2:**
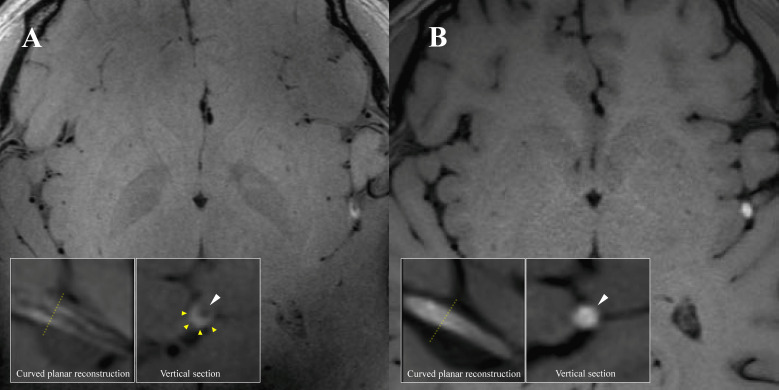
(A) 7T 3D T1-SPACE MRI (MAGNETOM Terra, Siemens Healthcare, Erlangen, Germany. Voxel size=0.4×0.4×0.4 mm^3^) showing left distant M2 segment MCA mural haematoma and residual lumen, and axial reformatted images showing a semilunar hyperintense signal caused by a mural haematoma (yellow triangle) and an eccentric hypointense signal attributable to the residual lumen (white triangle). (B) 3T 3D T1-SPACE MRI (MAGNETOM Prisma, Siemens Healthcare, Erlangen, Germany. Voxel size=0.54×0.54×0.54 mm^3^) showing left distant M2 segment MCA intraluminal thrombus formation and axial reformatted images showing only hyperintense signal (white triangle) with an unclear residual lumen. MCA, middle cerebral artery.

A 36-year-old man was admitted for acute right extremity weakness with multiple acute infarctions in the left middle cerebral artery (MCA) territory. 7T and 3T MRI scans were completed at 9 and 10 days after symptom onset (figure 1). There was susceptibility vessel sign, which represents red blood cell dominant intraluminal thrombus,^1 2^ at left distal M2 segment MCA on susceptibility-weighted imaging (figure 1B). 7T vessel wall MRI showed a typical feature of a long segment dissection at the left distal M2 segment of MCA with a mural haematoma and the residual lumen (figure 2A), which was not seen on 3T (figure 2B). Vessel wall MRI was a valuable tool for the diagnosis of intracranial artery dissection.^2 3^ This case indicated that 7T vessel wall MRI may provide better detail on the pathological change than traditional 3T MRI for diagnosing distal intracranial arterial dissection, the diagnose otherwise could be missed.
